# Predicted Role of NAD Utilization in the Control of Circadian Rhythms during DNA Damage Response

**DOI:** 10.1371/journal.pcbi.1004144

**Published:** 2015-05-28

**Authors:** Augustin Luna, Geoffrey B. McFadden, Mirit I. Aladjem, Kurt W. Kohn

**Affiliations:** 1 Laboratory of Molecular Pharmacology, National Cancer Institute, Bethesda, Maryland, United States of America; 2 Department of Bioinformatics, Boston University, Boston, Massachusetts, United States of America; 3 Applied and Computational Mathematics Division, National Institute of Standards and Technology, Gaithersburg, Maryland, United States of America; University of Michigan, UNITED STATES

## Abstract

The circadian clock is a set of regulatory steps that oscillate with a period of approximately 24 hours influencing many biological processes. These oscillations are robust to external stresses, and in the case of genotoxic stress (i.e. DNA damage), the circadian clock responds through phase shifting with primarily phase advancements. The effect of DNA damage on the circadian clock and the mechanism through which this effect operates remains to be thoroughly investigated. Here we build an *in silico* model to examine damage-induced circadian phase shifts by investigating a possible mechanism linking circadian rhythms to metabolism. The proposed model involves two DNA damage response proteins, SIRT1 and PARP1, that are each consumers of nicotinamide adenine dinucleotide (NAD), a metabolite involved in oxidation-reduction reactions and in ATP synthesis. This model builds on two key findings: 1) that SIRT1 (a protein deacetylase) is involved in both the positive (i.e. transcriptional activation) and negative (i.e. transcriptional repression) arms of the circadian regulation and 2) that PARP1 is a major consumer of NAD during the DNA damage response. In our simulations, we observe that increased PARP1 activity may be able to trigger SIRT1-induced circadian phase advancements by decreasing SIRT1 activity through competition for NAD supplies. We show how this competitive inhibition may operate through protein acetylation in conjunction with phosphorylation, consistent with reported observations. These findings suggest a possible mechanism through which multiple perturbations, each dominant during different points of the circadian cycle, may result in the phase advancement of the circadian clock seen during DNA damage.

## Author Summary

Many physiological processes are regulated by the circadian clock, and we are continuing to learn about the role of the circadian clock in disease. Research in recent years has begun to shed light on the feedback mechanisms that exist between circadian regulation and other processes, including metabolism and the response to DNA damage. A challenge has been to understand the dynamic nature of the protein interactions of these processes, which often involve protein modification as a means of communicating cellular states, such as damaged DNA. Here we have devised a model that simulates an alteration of the circadian clock that is observed during DNA damage response. A novel aspect of this model is the inclusion of SIRT1, a protein that regulates core circadian proteins through modification and helps to repress gene expression. SIRT1 is dependent on a metabolite regulated by the circadian clock and is depleted during DNA damage. In conjunction with a second form of protein modification, our results suggest that multiple forms of protein modification may contribute to the experimentally observed alterations to circadian function.

## Introduction

Circadian rhythms are biological oscillations occurring with an approximately 24-hour period affecting many processes. In mammals, these oscillations are centrally controlled in the brain by the suprachiasmatic nuclei (SCN). The SCN synchronizes the peripheral circadian clocks that exist in nearly every cell. Disruption of the circadian clock can lead to higher incidence of certain forms of cancer, and circadian timing can affect both the tolerability and efficacy of cancer therapeutics though the underlying mechanisms for these effects are still not well-understood [1,2]. Mutations of core circadian components in tumors can affect several properties of circadian oscillations, including: changes in amplitude, phase shifts, and period [3].

Investigation into the molecular components of the circadian clock has revealed much about how these biological rhythms function. In mammals, the core of the circadian clock is coordinated by four components that operate in a transcription-translation feedback loop. The positive (i.e. transcriptional activation) arm of the circadian clock involves a transactivating heterodimer complex composed of Brain and Muscle Arnt-Like protein-1 (BMAL1) and Circadian Locomotor Output Cycles Kaput (CLOCK) that induces the transcription of many genes; the current model and its simplifications are described in the Model section. Gene expression microarray analyses have shown that as much as 10% of an organism's transcriptome could be under circadian influence with expression exhibiting circadian oscillations; this value depends on experimental conditions and the tissue of origin [4]. The BMAL1/CLOCK transactivating complex operates on E-box regions of gene promoters. Additionally, CLOCK is an acetyltransferase involved in chromatin remodeling that is required for the proper operation of the circadian clock [5]. The negative (i.e. transcriptional repression) arm of the circadian clock involves the Cryptochrome (CRY1 and CRY2) and Period (PER1, PER2, and PER3) genes that act as inhibitors of the BMAL1/CLOCK transcription factor complex. CRY/PER heterodimers in the nucleus suppress CLOCK/BMAL1-mediated transcription completing the feedback loop, which then repeats to result in increased transcriptional activity as the levels of CRY/PER complex diminish [6]. The degradation of CRY/PER levels is partially triggered by CKI-epsilon (Casein Kinase I-epsilon) mediated phosphorylation, which marks the PER proteins for proteasomal degradation [7]. Period (PER) proteins have been shown to interact with ATM and CHK2, two key proteins involved in DNA damage response; the *Neurospora* ortholog for CHK2, PRD-4, has been shown to promote the phosphorylation of the PER protein analogue in *Neurospora*, FRQ [8,9].

Several studies show the existence of interplay between the pathways regulating circadian rhythms and those regulating DNA damage response. For example, disruptions to the core components can lead to alterations in DNA damage response pathways through altered expression patterns [10]. The reverse has also been observed, in that circadian oscillations can be reset by genotoxic stress [11,12]. Rat-1 fibroblasts were subjected to pulses of ionizing radiation resulting primarily in phase advancements of circadian oscillations [11]. In contrast, other forms of perturbation produce phase advancements and delays, such as in the case of pharmacological perturbation with dexamethasone [13]. Dexamethasone is a glucocorticoid agonist capable of resetting the circadian phase of asynchronous cells by triggering the expression of PER1 [14].

The molecular basis for the regulation of the circadian clock in the presence of genotoxic stress continues to be explored [11,12]. As our understanding of circadian regulation expands, so do the interconnections with other biological processes. Several recent studies have shown the circadian clock to be regulated by proteins, such as SIRT1, involved with DNA damage response and cellular metabolic state through their consumption of nicotinamide adenine dinucleotide (NAD) [15,16]. NAD participates in many oxidation-reduction reactions and functions, including ATP production [17]. Supplies of NAD are under circadian regulation due to circadian oscillation of nicotinamide phosphoribosyltransferase (NAMPT) that controls a rate-limiting step in the salvage of NAD [16,18].

In its DNA damage response role, NAD is involved in cell fate decisions through its utilization by PARP1 and SIRT1, as recently reviewed [19]. PARP1 is an ADP-ribosyltransferase where the ADP-ribosyl moieties are obtained from the cleavage of NAD. PARP1 is activated in the presence of DNA strand breaks (its activity can increase 10–500 fold) and helps to recruit DNA repair proteins [20,21]. At severe levels of DNA damage, energy depletion due to loss of NAD and ATP may trigger necrosis [20,22].

SIRT1 is an NAD-dependent protein deacetylase that can help inhibit transcription through histone deacetylation. The acetylation of histones leads to the activation of gene expression by inducing a relaxed chromatin confirmation at gene promoters, which permits the access of DNA transcription proteins [15]. Histone acetylation is counter-balanced through deacetylation causing a condensed chromatin state and transcriptional silencing. SIRT1 is involved in DNA damage responses through interaction with several key proteins, such as p53, where the deacetylation of p53 inhibits p53 and promotes cell survival [23]. More recently, SIRT1 has been implicated in the regulation of the circadian clock in several ways. First, SIRT1 destabilizes the interaction between CRY and BMAL1 through the deacetylation of BMAL1; the deacetylation of BMAL1 is counter-balanced at the same position through the acetyltransferase activity of CLOCK [15,24]. Second, SIRT1 has been shown to deacetylate PER destabilizing the protein and promoting its degradation, which may promote transcription during circadian oscillations [25]. Finally, SIRT1 is recruited to promoters of PER2 and NAMPT and is involved in the chromatin remodeling of the vicinity of each of the two promoters [16].

The circadian clock has been the subject of several mathematical models that have helped in our understanding of the molecular mechanisms underlying regulation of the circadian clock [26,27]. Our understanding of the NAD circadian regulation dynamics and the molecular mechanism regulating the phase resetting response of the circadian clock upon exposure to genotoxic stress remains incomplete; given the interactions mentioned above, it is possible that NAD utilization may be involved. We have developed an ordinary differential equation (ODE) model that includes the role of NAD in the regulation of SIRT1. The current study explores the potential role of NAD depletion in phase resetting of the circadian clock through the activities of the NAD consumers, SIRT1 and PARP1. Also, we examine the effect of multiple perturbations on the circadian cycle and how these perturbations may account for this observed behavior of the primarily phase advancement resetting of the circadian clock seen during DNA damage.

## Methods

### Model

We have developed a simple model (referred to here as the current model) representing the circadian clock of mammals, which extends a previous model developed by Hong et al. (referred to here as the Hong 2009 model) [28]. As in the Hong 2009 model, we only consider the activity of the PER protein and have subsumed the paralogs of the CRY (Cryptochrome) and PER (Period) genes into a single species CP in order to simplify the model. Within the model, PER can exist as a monomer, dimer, or in complex with BMAL1/CLOCK. BMAL1/CLOCK is inactivated when it exists in a complex with the PER dimer. Each form of PER contains a phosphorylation term that simulates the phosphorylation that triggers proteasomal degradation [7].

**Fig 1:**
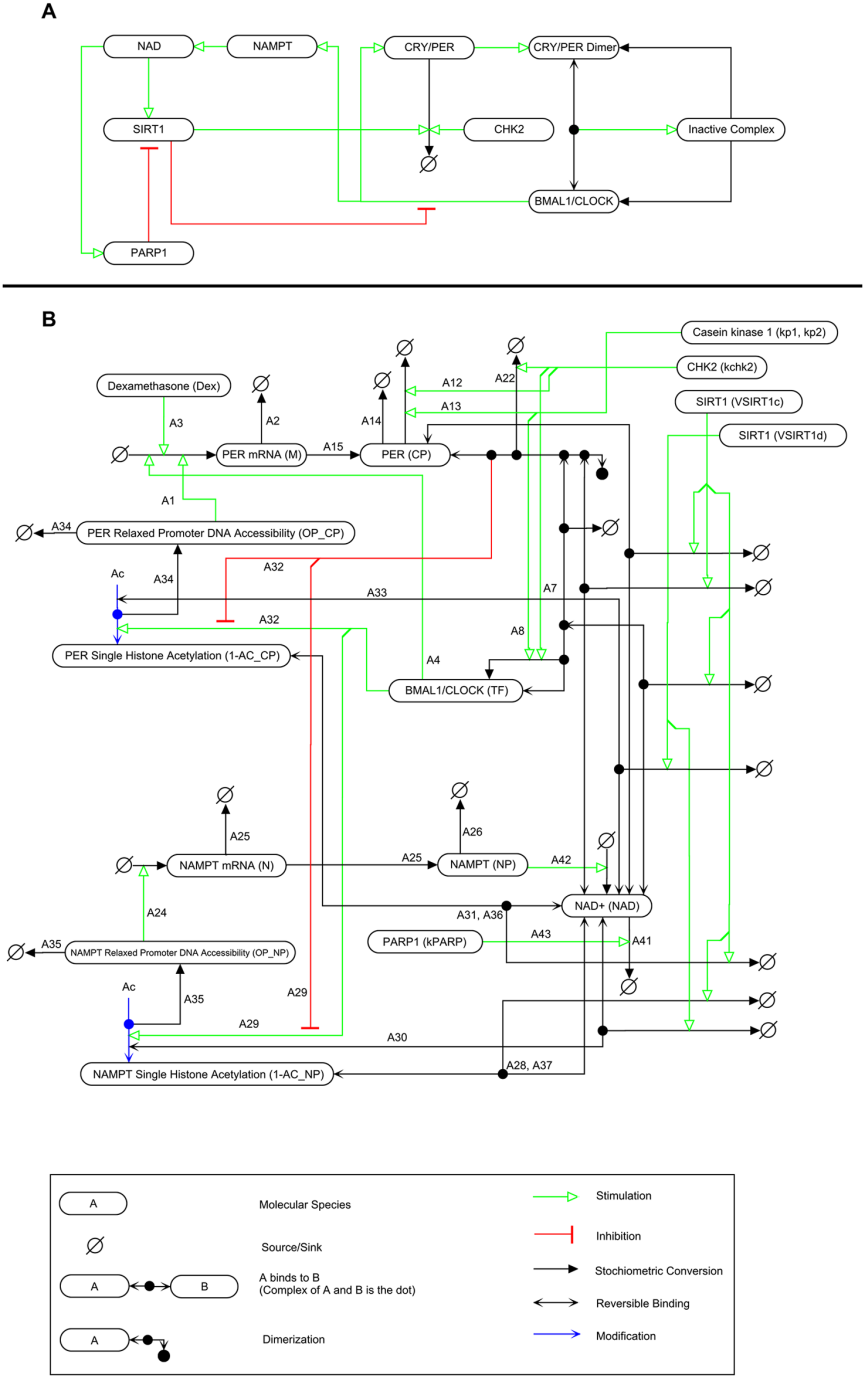
Molecular Interaction Map (MIM) wiring diagram of the simulated system. A) A simplified diagram highlighting the key activities of model species. BMAL1/CLOCK stimulates the production of both NAMPT and CRY/PER. CRY/PER dimerizes and binds to BMAL1/CLOCK to form an inactive complex. CHK2 stimulates the degradation of CRY/PER. NAMPT stimulates the production of NAD which in turn stimulates the activities of SIRT1 and PARP1. SIRT1 stimulates the degradation of CRY/PER and inhibits BMAL1/CLOCK transcriptional activity. PARP1 inhibits SIRT1 activity through consumption of NAD, and B) Full MIM representation of model.

Fig. 1 shows a wiring diagram for the current model using the Molecular Interaction Map (MIM) notation for bioregulatory networks and drawn using PathVisio-MIM [29,30]. Each interaction is labelled and described in Table 1; these descriptions are used to label the reactions in the SBML model file.

**Table 1:** Description of MIM wiring diagram and connection to model equations.

MIM Annotation	Description	Equation
A1	CP mRNA synthesis (OP_CP_)	1
A2	CP mRNA degradation	1
A3	CP mRNA synthesis (Dex induction)	1
A4	CP mRNA synthesis (TF)	1
A5	IC association	2
A6	IC degradation (phosphorylation)	2
A7	IC degradation (CHK2)	2
A8	IC degradation	2
A9	IC disassociation	2
A10	IC degradation (NAD)	2
A11	CP degradation (NAD)	3
A12	CP degradation (CHK2)	3
A13	CP degradation (phosphorylation)	3
A14	CP degradation	3
A15	CP synthesis	3
A16	CP2 degradation (NAD)	4
A17	IC association	4
A18	CP2 degradation (CHK2)	4
A19	CP2 degradation (phosphorylation)	4
A20	IC degradation	4
A21	IC disassociation	4
A22	CP2 disassociation	4
A23	CP dimerization	4
A24	N mRNA synthesis (OP_NP_)	5
A25	N degradation	5
A26	NP degradation	6
A27	NP synthesis	6
A28	NP acetylated histone deacetylation (NAD)	7
A29	AC_NP_ histone acetylation	7
A30	NP acetylated histone deacetylation	7
A31	CP acetylated histone deacetylation (NAD)	8
A32	AC_CP_ histone acetylation	8
A33	CP acetylated histone deacetylation	8
A34	OP_CP_ synthesis	9
A35	OP_NP_ synthesis	10
A36	CP acetylated histone deacetylation (NAD)	11
A37	NP acetylated histone deacetylation (NAD)	11
A38	IC degradation (NAD)	11
A39	CP2 degradation (NAD)	11
A40	CP degradation (NAD)	11
A41	NAD degradation	11
A42	NAD synthesis	11
A43	NAD degradation (PARP1)	11
A44	CP2 disassociation	3
A45	CP dimerization	3

The description column contains species labels from Table 3; IC refers to the “inactive complex” from Equation 14.

The original form of CRY/PER mRNA transcription in the Hong 2009 model used a Hill function, but this is zeroed out in the current model using *kms (kms = 0)* in Equation 1 (below). We extend the Hong 2009 model to account for the effects of acetylation on transcription for both PER and NAMPT by using Equation 1 through Equation 15 from Smolen et al. re-worked for the system described in the current model; these equations become the method for describing transcription rather than usage of a Hill function [31]. Deacetylation of histones results in chromatin compaction and decreased transcription as a result of lowered accessibility of DNA polymerase to these regions of condensed chromatin. In the case of PER, the first term of Equation 8 accounts for the fractional levels of histone acetylation. The rate of promoter acetylation is a function of acetylation regulated by the BMAL1/CLOCK (TF) complex through CLOCK acetyltransferase activity and inhibited by the effects PER dimer, Equation 13. Further, it is known that CLOCK is able to acetylate histones at positions deacetylated by SIRT1 [15]. The rate of histone acetylation is regulated by the basal rate of histone deacetylation and the SIRT1 deacetylation activity simulated as a two substrate Michaelis-Menten reaction that utilizes NAD in the process; the activity of SIRT1 is discussed further below. Therefore, unlike Smolen et al., we do not use a single, fixed deacetylation rate [31]. This is consistent with the work of Nakahata et al., which showed that peak SIRT1 deacetylation activity coincided with the lowest acetylation levels of histone H3 [15]. This level of single histone acetylation is then used to generate an overall promoter accessibility value, Equation 9. Lastly, this promoter accessibility value is multiplied by a maximal rate of transcription to denote the expression of PER, Equation 1. The same mechanism is used to denote the expression of NAMPT.

Neither SIRT1 expression nor protein levels are under circadian control, yet its deacetylation activity is regulated in a circadian manner [15]. Therefore, we do not consider changes to SIRT1 levels and only consider the ability of SIRT1 to utilize NAD to deacetylate three species (PER, BMAL1/CLK, and acetylated histone) within the model, thereby affecting circadian rhythms via separate mechanisms. First, SIRT1 deacetylates PER2 destabilizing the protein and promoting its degradation [25]. Second, acetylation of BMAL1 promotes the binding of CRY1 to BMAL1 and BMAL1 is a target of SIRT1 deacetylation [32]. Third, as a histone deacetylase SIRT1 is able to deacetylate lysine residues of histones helping to produce transcriptionally silenced chromatin that exists with a closed chromatin structure [33]. Two parameters specify the activity of SIRT1 in the model. The first parameter VSIRT1c regulates the deacetylation of PER (either monomer, dimer, or in complex with BMAL1/CLOCK) and the second parameter, VSIRT1d, regulates the histone deacetylation. The levels of NAD are regulated using a first-order reaction dependent on the availability of NAMPT. The model includes perturbation inputs from the Hong 2009 model, dexamethosone (*Dex*) and the CHK2 phosphorylation (*kchk2* affecting PER monomer and dimer and *kchk2c* affecting PER in complex with BMAL1/CLOCK).

All simulations were conducted using MATLAB (http://www.mathworks.com). Copies of our model as a Systems Biology Markup Language (SBML) generated using COPASI (http://www.copasi.org) are published as supporting information on the PLOS website (S1 File).

### Kinetic equations

The model is a system of 11 equations described above and shown below. Equation 12 and Equation 13 denote the rate promoter acetylation for the NAMPT and PER promoters, respectively. Equation 14 denotes the level of inactive complex, while Equation 15 is the total amount of PER that exists in the system.

Equation 1: CRY/PER mRNA

$$\frac{dM}{dt}=VM\cdot OP_{CP}-k_{md}\cdot M+Dex+\frac{k_{ms}\cdot TF^{n}}{J^{n}+TF^{n}}$$

Equation 2: BMAL1/CLOCK complex

$$\frac{dTF}{dt}=-k_{ica}CP2\text{⋅}TF+\frac{k_{p2}IC}{J_{p}+CP_{tot}}+k_{chk2c}\text{⋅}IC+k_{cp2d}IC+k_{icd}IC-\frac{V_{SIRT1c}IC\text{⋅}NAD}{k_{aCPSIRT1}\text{⋅}k_{bCPSIRT1}+k_{bCPSIRT1}\text{⋅}IC+IC\text{⋅}NAD}$$

Equation 3: CRY/PER protein monomer

$$\frac{dCP}{dt}=-\frac{V_{SIRT1c}CP\text{⋅}NAD}{k_{aCPSIRT1}\text{⋅}k_{bCPSIRT1}+k_{bCPSIRT1}\text{⋅}CP+CP\text{⋅}NAD}-k_{chk2}CP-\frac{k_{p1}\text{⋅}CP}{J_{p}+CP_{tot}}+2k_{d}CP2-2k_{a}CP\text{⋅}CP-k_{cpd}CP+k_{cps}M$$

Equation 4: CRY/PER protein dimer

$$\frac{dCP2}{dt}=-\frac{V_{SIRT1c}CP2\text{⋅}NAD}{k_{aCPSIRT1}\text{⋅}k_{bCPSIRT1}+k_{bCPSIRT1}\text{⋅}CP2+CP2\text{⋅}NAD}-k_{ica}CP2\text{⋅}TF-k_{chk2}CP2-\frac{k_{p2}CP2}{J_{p}+CP_{tot}}-k_{cp2d}CP2+k_{icd}IC-k_{d}CP2+k_{a}CP\text{⋅}CP$$

Equation 5: NAMPT mRNA

$$\frac{dN}{dt}=VN\text{⋅}OP_{NP}-k_{nd}N$$

Equation 6: NAMPT protein

$$\frac{dNP}{dt}=-k_{npd}NP+k_{nps}N$$

Equation 7: Single histone acetylation (NAMPT promoter)

$$\frac{dAC_{NP}}{dt}=-\frac{V_{SIRT1d}AC_{NP}NAD}{k_{aCPSIRT1}k_{bCPSIRT1}+k_{bCPSIRT1}AC_{NP}+AC_{NP}NAD}-k_{np_{ac}}*(1-AC_{NP})-k_{Np_{deac}}*AC_{NP}$$

Equation 8: Single histone acetylation (CRY/PER promoter)

$$\frac{dAC_{CP}}{dt}=-\frac{V_{SIRT1d}AC_{CP}NAD}{k_{aCPSIRT1}k_{bCPSIRT1}+k_{bCPSIRT1}AC_{CP}+AC_{CP}NAD}+k_{cp_{ac}}*(1-AC_{CP})-k_{cp_{deac}}*AC_{CP}$$

Equation 9: DNA accessibility value (CRY/PER promoter)

$$\frac{dOP_{CP}}{dt}=\frac{\left(AC_{CP}{}^{n_{ac}}-OP_{CP}\right)}{T_{const\_cp}}$$

Equation 10: DNA accessibility value (NAMPT promoter)

$$\frac{dOP_{NP}}{dt}=\frac{\left(AC_{NP}{}^{n_{ac}}-OP_{NP}\right)}{T_{const\_np}}$$

Equation 11: NAD

$$\begin{array}{l} \frac{dNAD}{dt}=-\frac{V_{SIRT1d}AC_{CP}NAD}{k_{aCPSIRT1}k_{bCPSIRT1}+k_{bCPSIRT1}AC_{CP}+AC_{CP}NAD}-\frac{V_{SIRT1d}AC_{NP}NAD}{k_{aCPSIRT1}k_{bCPSIRT1}+k_{bCPSIRT1}AC_{NP}+AC_{NP}NAD}\\ -\frac{V_{SIRT1c}ICNAD}{k_{aCPSIRT1}k_{bCPSIRT1}+k_{bCPSIRT1}IC+IC\text{·}NAD}-\frac{V_{SIRT1c}CP2\text{·}NAD}{k_{aCPSIRT1}k_{bCPSIRT1}+k_{bCPSIRT1}CP2+CP2\text{·}NAD}\\ -\frac{V_{SIRT1c}CP\text{·}NAD}{k_{aCPSIRT1}k_{bCPSIRT1}+k_{bCPSIRT1}CP+CP\text{·}NAD}-k_{nadd}NAD+VNADcNP-k_{PARP}NAD \end{array}$$

Equation 12: Rate of NP promoter acetylation

$$k_{npac}=\frac{TF}{TF+K_{TFNP}}\text{⋅}\frac{K_{CP2NP}}{K_{CP2NP}+CP2}$$

Equation 13: Rate of CP promoter acetylation

$$k_{cpac}=\frac{TF}{TF+K_{TFCP}}\text{⋅}\frac{K_{CP2CP}}{K_{CP2CP}+CP2}$$

Equation 14: Inactive complex (BMAL1/CLOCK and PER dimer)

$$IC=TF_{tot}-TF$$

Equation 15: Total amount of PER

$$CP_{tot}=CP+2CP2+2IC$$

### Kinetic parameters

Kinetic parameters used for the current model are described in Table 2; the table also lists the parameter values necessary to reconstitute the Hong 2009 model. Rate constants were based on previously published circadian models [28,31]. Kinetic parameters unique to the current model were then optimized to generate oscillations in the current work. Rate constants are in units of h^-1^. The resulting amplitudes have similar orders of magnitude to the original Hong 2009 model.

**Table 2:** Parameter values for current and Hong 2009 model.

Parameter	Description	Current Model	Hong 2009
Dex	Rate of CRY/PER mRNA synthesis by dexamethasone	0	0
k_ms_	Rate of CRY/PER mRNA synthesis	0	1
J	Michaelis constant for BMAL1/CLOCK binding to CRY/PER promoter	0	0.3
k_md_	Rate of CRY/PER mRNA degradation	0.13857	0.1
k_cps_	Rate of CRY/PER protein synthesis	0.40453	0.5
k_cpd_	Rate of CRY/PER protein degradation	0.48936	0.525
k_a_	Rate of CRY/PER dimer association	49.9712	100
k_d_	Rate of CRY/PER dimer disassociation	0.36005	0.01
k_p1_	Rate for monomer phosphorylation	9.4531	10
J_p_	Michaelis constant of protein kinase (Casein Kinase 1 Epsilon, CSNK1E)	77.9254	0.05
k_chk2_	Rate of phosphorylation by CHK2	0	0
k_icd_	Rate of inactive complex (BMAL1/CLOCK/(CRY/PER)/(CRY/PER)) degradation	0	0.01
k_cp2d_	Rate of CRY/PER dimer degradation	0.0025	0.0525
k_ica_	Rate of inactive complex association	28.178	20
k_chk2c_	Rate of phosphorylation by CHK2	0	0
k_p2_	Rate for dimer phosphorylation	0.36296	0.1
Tf_tot_	Total amount of BMAL1/CLOCK	0.84792	0.5
K_TFCP_	Binding affinity of BMAL1/CLOCK to CRY/PER promoter	0.020133	0
K_CP2CP_	Binding affinity of CRY/PER dimer to CRY/PER promoter	0.20757	0
K_TFNP_	Binding affinity of BMAL1/CLOCK to NAMPT promoter	0.040267	0
K_CP2NP_	Binding affinity of CRY/PER dimer to NAMPT promoter	0.21591	0
k_cpdeac_	Rate of CRY/PER promoter deacetylation	0.099	0
k_npdeac_	Rate of NAMPT promoter deacetylation	0.098073	0
VM	Rate of CRY/PER expression	0.40053	0
VN	Rate of NAMPT expression	0.56383	0
n	Hill coefficient for CRY/PER mRNA synthesis	0	2
n_ac_	Value used to describe the steady state values for promoter accessibility	1.6107	0
T_const_np_	Time constant for the relaxation of NAMPT promoter to steady state value	0.26014	1
T_const_cp_	Time constant for the relaxation of CRY/PER promoter to steady state value	0.22107	1
VSIRT1c	Rate of SIRT1 activity (non-histone deacetylation)	0.094568	0
k_nadd_	Rate of NAD+ degradation	1.3309	0
k_nd_	Rate of NAMPT mRNA degradation	0.16337	0
k_nps_	Rate of NAMPT protein synthesis	0.20238	0
k_npd_	Rate of NAMPT protein degradation	0.16024	0
VNADc	Rate of NAD+ production	5.2479	0
k_aCPSIRT1_	Disassociation constant of SIRT1 and NAD+ (Used with non-histone-related equations)	0.10491	0
k_bCPSIRT1_	Michaelis constant for non-histone substrates	0.098395	0
k_PARP_	Rate of PARP1 activity	0	0
VSIRT1d	Rate of SIRT1 activity (histone deacetylation)	0.070926	0

### Initial values

Initial values used in the current model are described in Table 3; initial values to reconstitute the Hong 2009 model are also listed in Table 3. The concentrations of proteins and metabolites are in arbitrary units (AU) because these are currently not known for many circadian clock proteins.

**Table 3:** Initial values for the current and the Hong 2009 model.

Species	Description	Current Model	Hong 2009 Model
M	CRY/PER mRNA	1.4	1.4
TF	BMAL1/CLOCK complex	0.13	0.13
CP	CRY/PER protein	0.037	0.037
CP2	CRY/PER dimer	0.046	0.046
N	NAMPT mRNA	1.5	1.5
NP	NAMPT protein	1	1
AC_NP_	Single histone acetylation (NAMPT) promoter)	0	0.01
AC_CP_	Single histone acetylation (CRY/PER) promoter)	0	0.01
OP_CP_	DNA Accessibility Value (CRY/PER) promoter)	0	0
OP_NP_	DNA Accessibility Value (NAMPT) promoter)	0	0
NAD	NAD+	3	3

### Simulation of DNA damage response

Damage was simulated by altering levels of *k*_*parp*_ and *k*_*chk2*_ as described in the Results section using the parameters in Table 4.

**Table 4:** Results for the analysis of phase shifting behavior by variables kchk2 and kparp.

Model	kchk2 Value	kparp Value	Maximum Delay (h)	Maximum Advance (h)	Advance to Delay Ratio	Positive Area Fraction
Hong 2009	0.2	0	-1.6766	5.0623	3.0193	0.8513
Current	0.2	0	-0.4922	3.6996	7.5161	0.918
Current	0	10	-0.9123	3.0428	3.3353	0.6814
Current	0	20	-1.6618	4.9971	3.0070	0.6235
Current	0.1	10	-0.0612	2.7985	45.7412	0.9966
Current	0.1	20	-0.6026	4.6307	7.6845	0.8538
Current	0.2	10	-0.0848	2.5542	30.1066	0.9979
Current	0.2	20	-0.1883	4.2642	22.6498	0.9857

### Period calculation

The period was calculated by finding the mean of the simulated results and then finding the time points where a selected time point was greater than the mean and the subsequent time point was less than the mean. For each of the selected time points, the previous time point was subtracted to produce the period value. The resulting values were then averaged for the final period value; a requirement was imposed that at least seven oscillations were necessary to produce this value otherwise an error value, negative one, was produced. The period was calculated using the time series for the CRY/PER (CP) protein.

### Phase Response Curve (PRC) calculation

Differences in phase were calculated after 19 days (19 circadian oscillations) between the unperturbed and perturbed systems. The phase shift (advancement or delay) was calculated using the difference between oscillation peaks for the two systems. Treatments were induced at each circadian hour, and the phase response curve was calculated using the time series data for the CRY/PER (CP) protein.

## Results

### Comparison of simulated oscillations to previous experimental results

**Fig 2:**
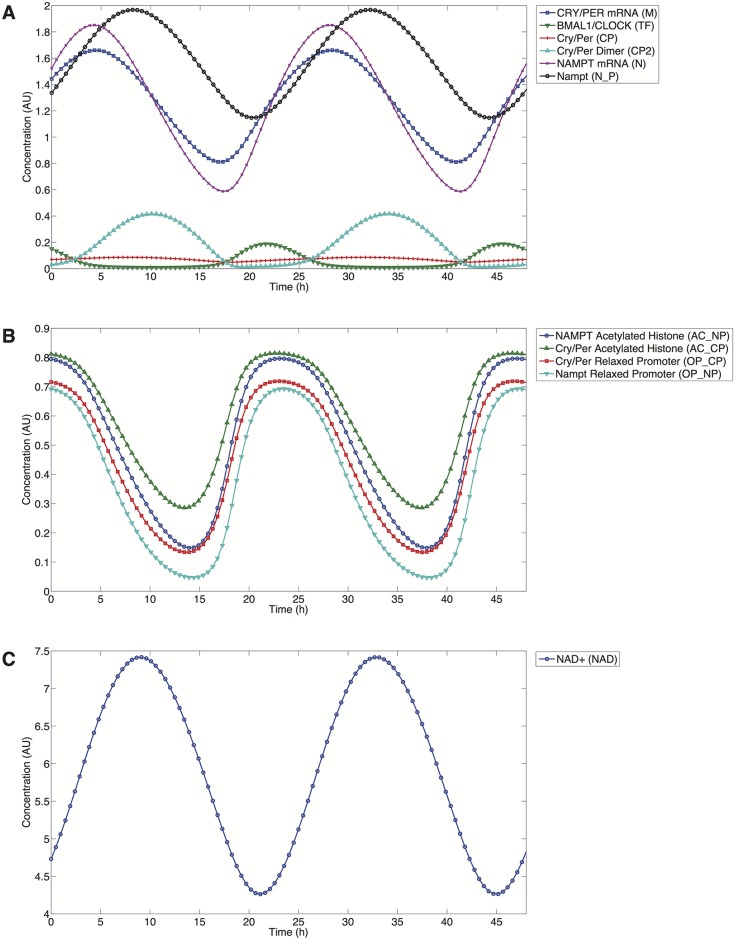
Current model time series. A) Main components: Blue/Square: CRY/PER mRNA, Green/Down Triangle: BMAL1/CLOCK, Red/Plus: CRY/PER, Cyan/Up Triangle: CRY/PER Dimer, Purple/X-Mark: NAMPT mRNA, Black/Circle: NAMPT protein. B) Histones and relaxed chromatin. Blue/Circle: NAMPT single histone acetylation levels, Green/Up Triangle: CRY/PER single histone acetylation levels, Red/Square: CRY/PER DNA accessibility value for relaxed promoter, Cyan/Down Triangle: NAMPT DNA accessibility value for relaxed promoter. C) NAD. Blue/Circle: NAD concentration. Parameters taken from Table 2 for the current model).

Fig. 2A illustrates the oscillatory behavior simulated by the model for the core circadian components using the current parameter set outlined in Table 2. The system oscillates with an autonomous period of 23.8 hours, which is well within the range seen in circadian oscillations of mice [34]. The current model simulates a free-running circadian clock without external stimuli or cues (zeitgebers) periodically synchronizing the clock and this is the state in which current model results are described. The model can account for entrainment by varying the *Dex* as a square-wave increasing the value of *Dex* to 0.125 for 12 hours and decreasing it to 0 for another 12 hours. Circadian models, such as the one by Leloup and Goldbeter in 2003, make use of varying PER transcription to simulate the effect of light entrainment. Dexamethasone with its ability to trigger PER transcription therefore is a suitable substitute for entrainment by light [14,35].

Fig. 2B illustrates the oscillations in the histone acetylation levels for both PER and NAMPT mRNA. Histone acetylation levels peak at approximately hour 22 in Fig. 2B, helping the relaxation of DNA to permit transcription to be initiated. The peak levels of PER and NAMPT mRNA are then reached after a lag of ~6 hours. Experimentally, peaks in the acetylation levels of histones H3 and H4 have been observed 4 and 8 hours in advance of the PER1 and PER2 mRNA peaks [36]. Acetylated histone and NAD levels oscillate in antiphase, as seen when comparing Fig. 2B and Fig. 2C. In the context of the model, this is due to a feedback mechanism involving NAD production and SIRT1 activity where NAD levels (NAD) rise to their peak measured levels ~5 hours after the peak levels of NAMPT mRNA (N). This is the time when SIRT1 activity is at its maximum and acetylated histone levels decline to their minimum ~5 hours later.

NAD levels oscillate by approximately 40% during each circadian cycle, as shown in Fig. 2C, in response to oscillations in NAMPT protein levels; NAD levels oscillate in phase with NAMPT levels. Similar changes in oscillations levels have been seen experimentally [16,18]. This decline in the NAD levels is a product of several SIRT1 deacetylation processes captured by the current model, as well as the basal degradation of NAD levels via processes external to the model.

**Fig 3:**
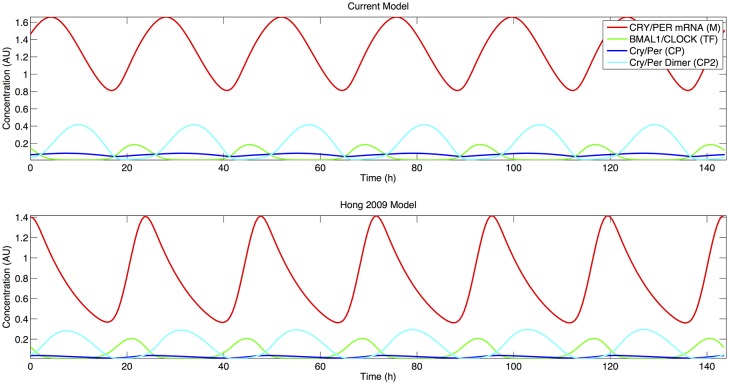
Comparison of current (Top) and Hong 2009 (Bottom) models. Red: CRY/PER mRNA, Green: BMAL1/CLOCK, Blue: CRY/PER protein, Cyan: CRY/PER Dimer. Only species common to both models are included.

Fig. 3 shows that the current model retains the phase dynamics present in the Hong 2009 model that are critical in the modeling of circadian systems. There is a lag of ~3 hours between the peak of PER mRNA and the peak in PER monomer levels; this is similar to experimental results seen for mammalian circadian rhythms [6]. Peaks in the PER monomer levels then proceed prior to the peak in the PER dimer levels several hours later, and peak levels in the PER dimer are then antiphase to the levels of the transcription factor BMAL1/CLOCK.

The Hong 2009 model possesses an autocatalytic positive feedback loop involving PER that is necessary to sustain oscillations [28]. This feedback loop requires that differential stabilities exist between PER monomer and PER in complexes, either the dimeric form alone or in the dimeric form complexed with BMAL1/CLOCK. This mechanism arises from experimental evidence in the Drosophila circadian clock by Kloss et al. wherein PER complexes were shown to be less susceptible to degradation [37]. The current model exhibits the same autocatalytic requirement with a smaller value for the degradation of the PER dimer (*kcp2d*) than for the degradation of the monomeric PER form (*kcpd*) by two magnitudes of order. In contrast to the Hong 2009 model which possesses values for the two parameters (*kcpd* and *kcp2d*) with a smaller difference, in the current model we assume the activity of SIRT1 (*VSIRT1c*) in the destabilization of PER in either monomeric or in complexes to be equivalent, which means that *kcpd2d* accounts for a smaller portion of the degradation of the PER dimer.

### Model robustness to parameter variation

Due to the importance of circadian rhythms in the synchronization of biological processes, circadian oscillations must be robust to minor perturbations and must stably oscillate in the presence of varied parameters resulting from individual variation. The results of a study of the circadian rhythms of 72 mice from 12 inbred mouse strains showed this robustness of circadian oscillations [34]. Across the combined strains, the period mean was 23.53 (range 22.94 to 23.93) hours. We expected a similar robustness in the current model and tested the sensitivity of the model to perturbations of each parameter individually using a method that has been used in computational studies previously [31,38].

**Fig 4:**
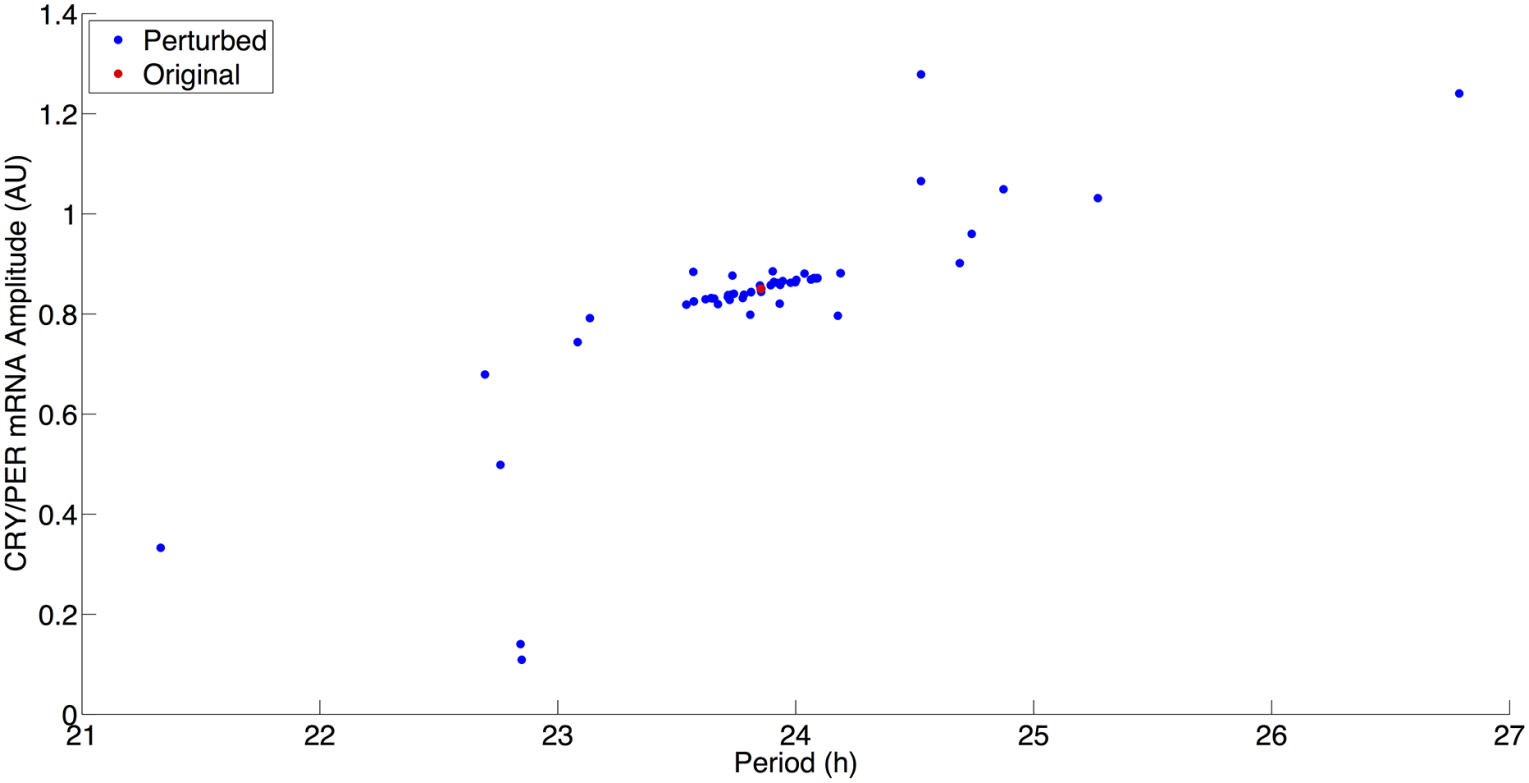
Model robustness as indicated by alterations in maximum peak-to-peak amplitude and period. Red: Current model parameter values. Blue: Perturbed parameter values individually increased and decreased by 20%.

Model robustness was tested by increasing and decreasing parameter values individually by 20% and plotting the resulting amplitude changes in PER mRNA (often used as an experimental proxy in PER luciferase experiments) against the oscillation periods. The results of this testing are shown in Fig. 4, and this testing suggests that the model is robust to parameter perturbations. Out of the perturbations tested, none of the parameter sets resulted in periods that deviated from 24 hours by more than 3 hours. A majority of the parameter perturbations clustered near the current model parameter values from Table 2 (this is shown in red in Fig. 4) with only slight increases or decreases of the period and amplitude. Stress input variables: *Dex*, *kchk2*, *kchk2c*, and *kPARP* are set to 0 in the current model parameter set, and therefore, they are not expected to, nor did they, have any effect during the sensitivity testing.

Three parameters resulted in periods less than 23 hours and PER mRNA amplitudes less than 0.4 AU. All three of these parameters affected PER, either mRNA or protein, levels. Decreases of 20% to PER protein synthesis rate (*kcps*) and PER mRNA synthesis rate (*VM*), resulted in this behavior, while an increase of 20% to the PER mRNA degradation *(kmd)* also resulted in a similar behavior with a decreased amplitude and period. A 20% decrease in PER mRNA degradation resulted in the opposite behavior with both an increase in amplitude and a period; as shown in Fig. 4, this is the only parameter that resulted in periods greater than 26 hours.

**Fig 5:**
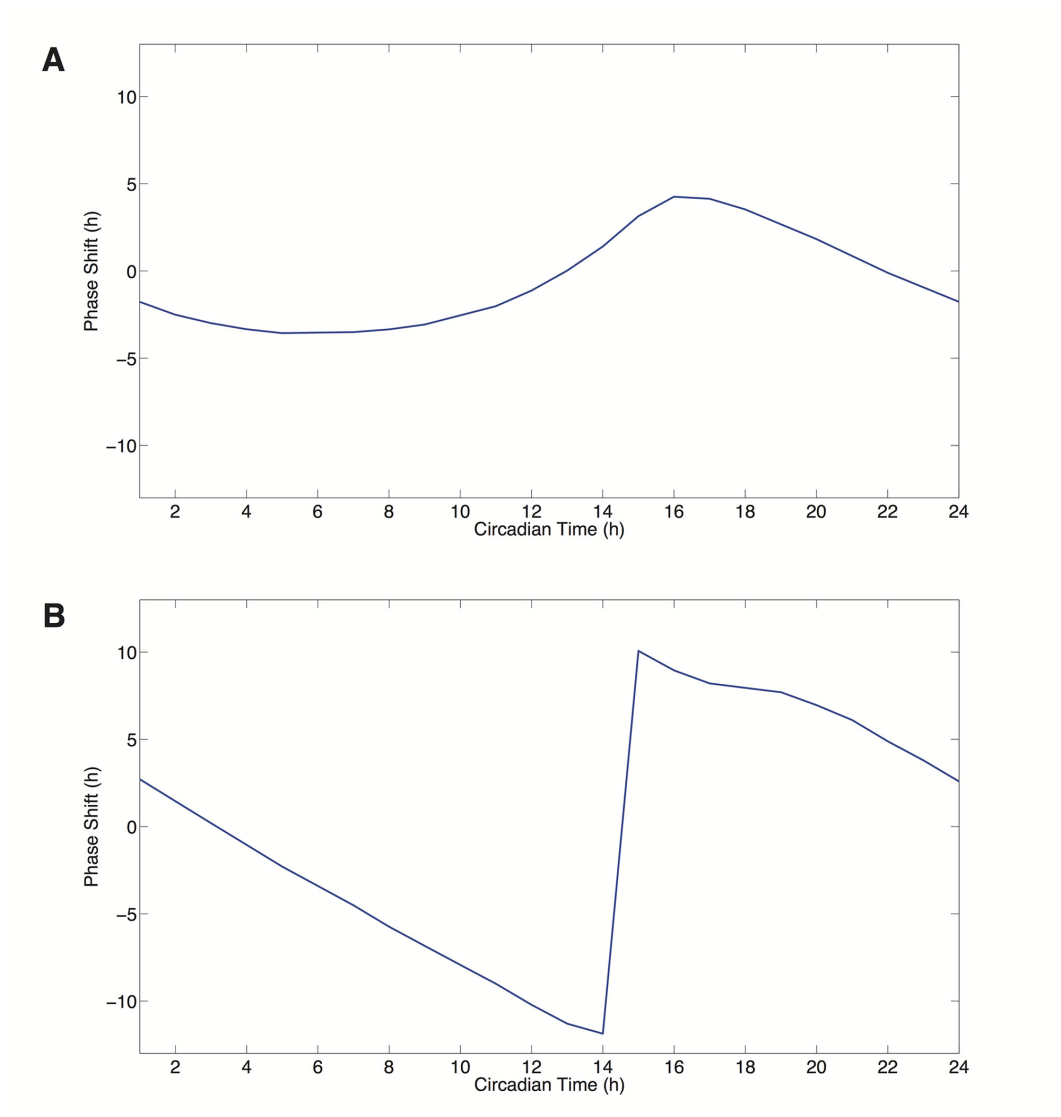
Model PRCs. A) Type 1 PRC. B) Type 0 PRC.

Next, phase response curves (PRCs) were generated using pulses of dexamethasone (*Dex*) which trigger the transcription of PER to show that the current model is able to produce both Type 1 and Type 0 PRCs as with the Hong 2009 model. Phase response curves illustrate the relationship between the timing of a perturbation and the effect of the perturbation on a circadian oscillation in the form of a phase shift [39]. There are two types of PRCs, Type 1 and Type 0. The resulting PRC is often dependent on the strength of the perturbation with Type 1 PRCs occurring at lower perturbations than Type 0. As shown in Fig. 5B, low values of *Dex* (*Dex* = 0.15) result in a Type 1 PRC (shown in Fig. 5A) whereby there is a continuous transition between phase advancements (positive values on the PRC) and delays (negative values) in response to the dexamethasone stimulus. At high values of *Dex* (*Dex* = 20), a Type 0 PRC is produced with a discontinuity between the phase advancements and delays of the system.

### Effect of NAD biosynthesis and SIRT1 activity on circadian rhythms

**Fig 6:**
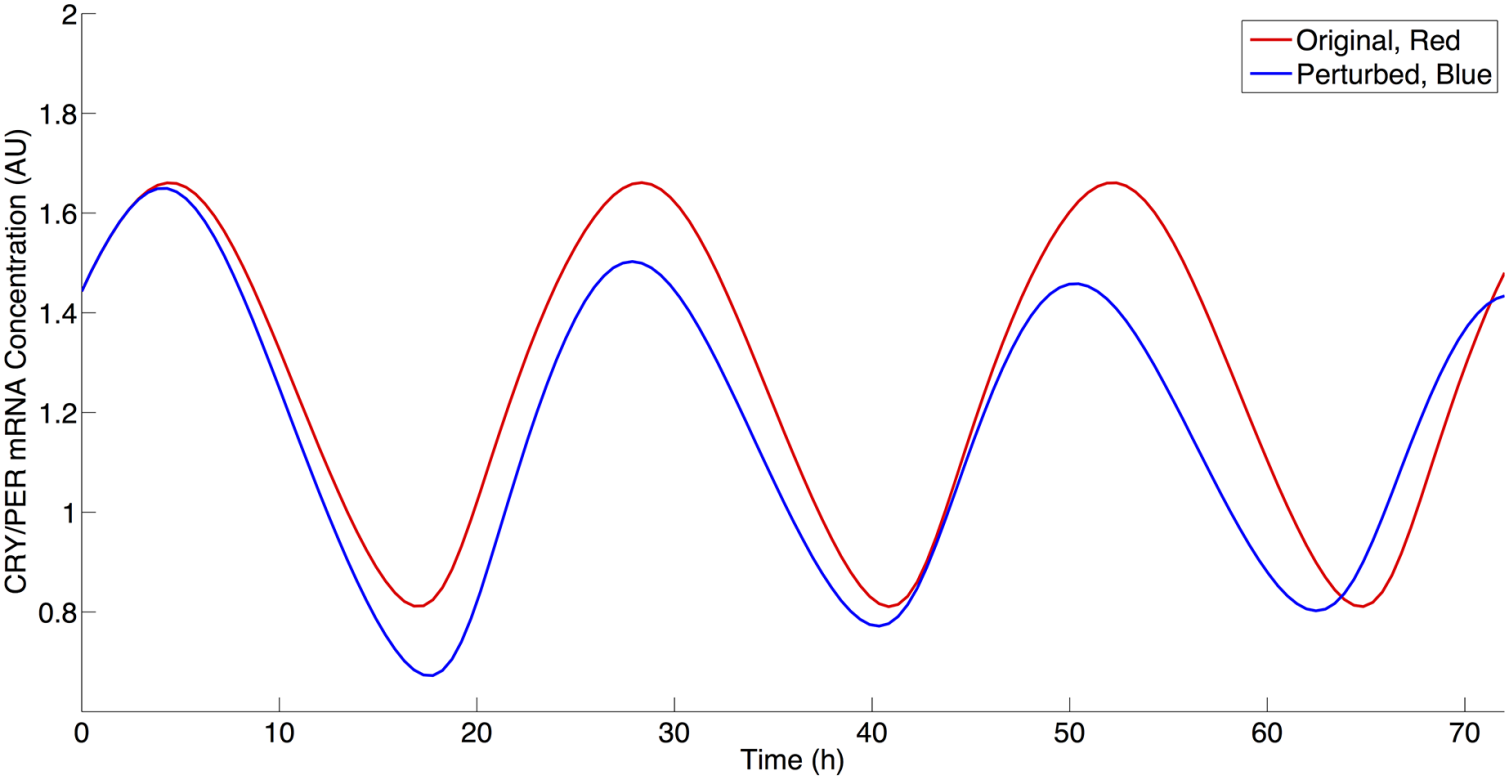
The effect of NAD biosynthesis decrease on CRY/PER mRNA levels. Red: Simulation using parameter values from Table 2. Blue: Simulation using parameter values from Table 2 with a 75% decrease in VNADc value.

**Fig 7:**
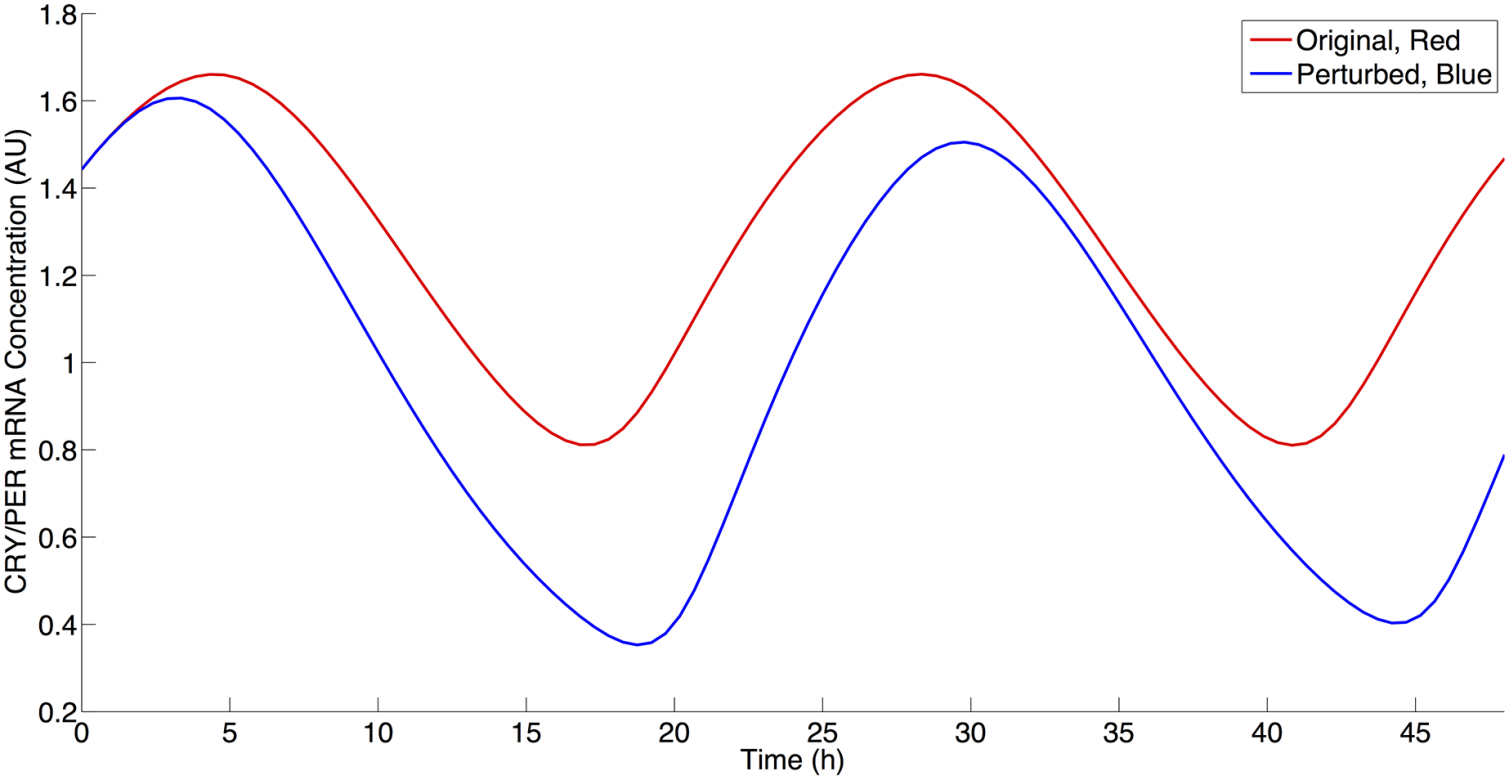
The effect of SIRT1 inhibition on non-histone components on CRY/PER mRNA levels. Red: Simulation using parameter values from Table 2. Blue: Simulation using parameter values from Table 2 with a 25% decrease in VSIRT1c value.

**Fig 8:**
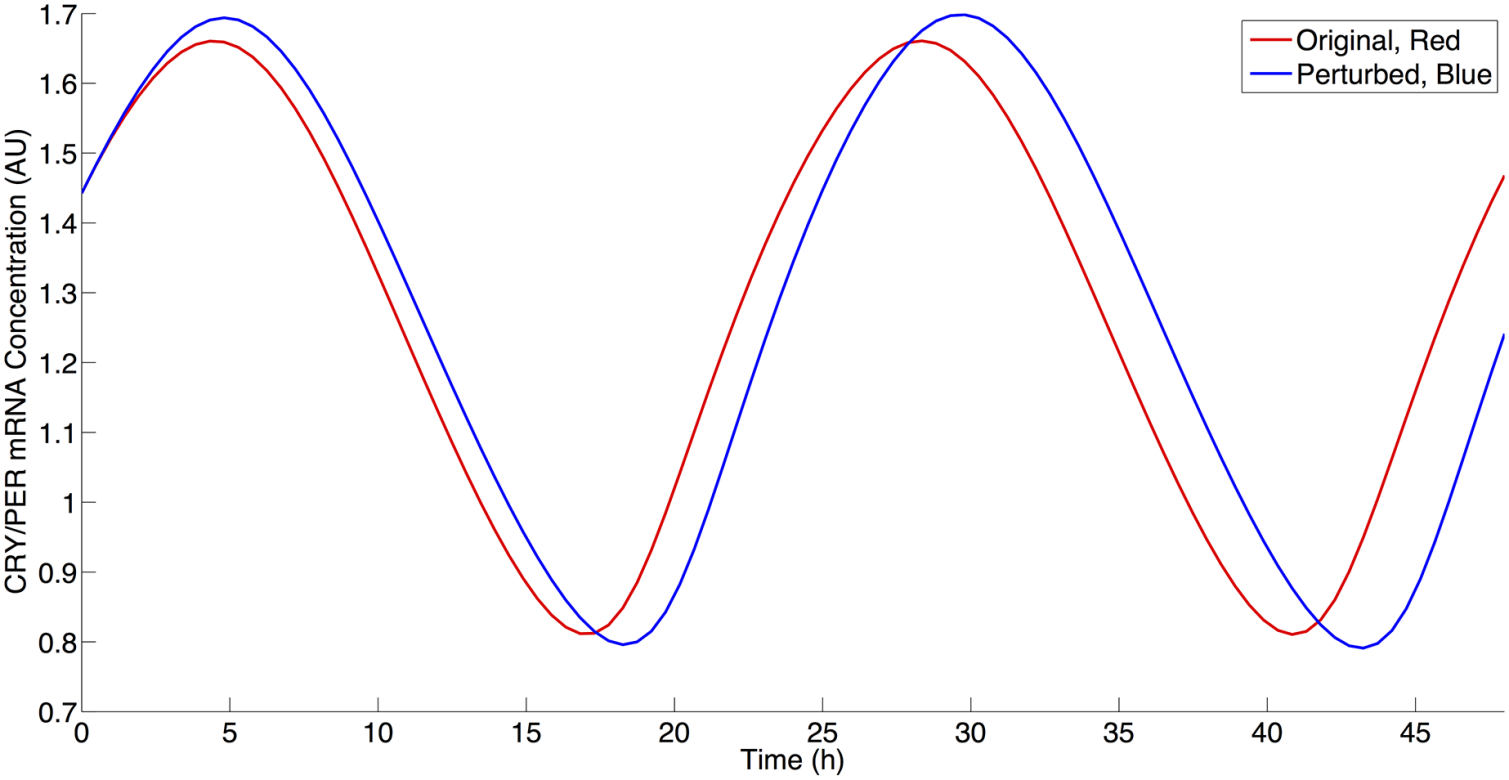
The effect of SIRT1 inhibition on histone components on CRY/PER mRNA levels. Red: Simulation using parameter values from Table 2. Blue: Simulation using parameter values from Table 2 with a 25% decrease in VSIRT1d value.

**Fig 9:**
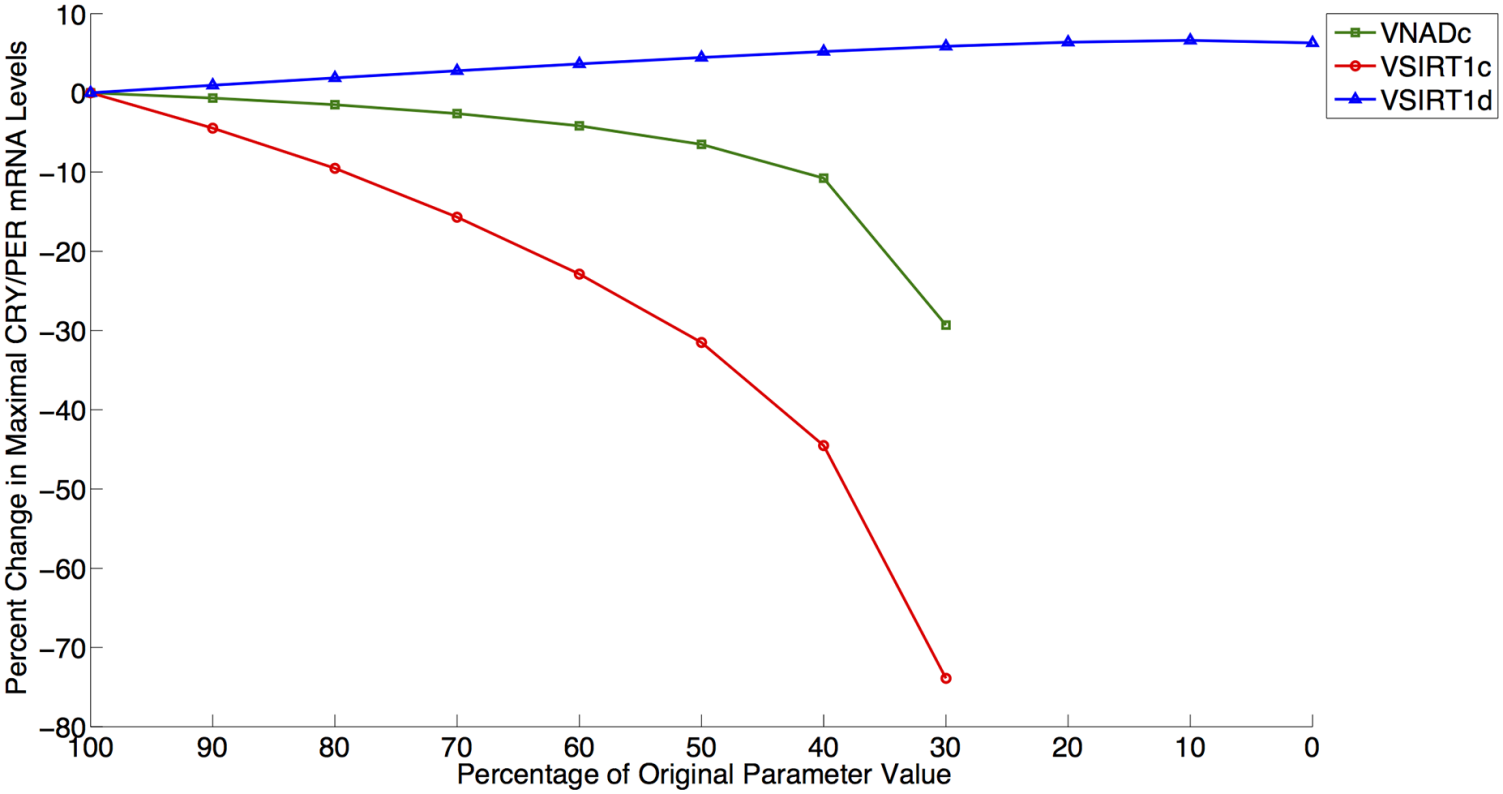
Percentage change in maximal levels of CRY/PER mRNA levels for SIRT1-related parameter values. Green/Square: VNADc; Red/Circle: VSIRT1c; Blue/Triangle: VSIRT1d. Data points shown for region of stable oscillation.

We next examined the roles of NAD biosynthesis and SIRT1 activity in the current model given the multiple deacetylation interactions in the model utilizing NAD via SIRT1 activity. Current literature contains a contradiction as to the effect of SIRT1 inhibition on PER2 mRNA levels. Nakahata et al. have shown that the inhibition of SIRT1 activity leads to an increased maximal level of PER2 mRNA [15,16]. Asher et al. have shown the reverse—that an inhibition SIRT1 activity results in a decrease in PER2 mRNA levels [25]. Both increases and decreases may be theoretically possible via SIRT1 activity, since SIRT1 can affect the positive (i.e. transcriptional activation) and negative (i.e. transcriptional repression) regulation arms of circadian rhythms. We began to address this apparent contradiction in our simulations by decreasing the rate of NAD biosynthesis. As shown in Fig. 6, this result agreed with the Asher et al. experimental results by qualitatively producing a decrease of approximately 12% in CRY/PER mRNA (M) levels following a decrease of 75% from the original *VNADc* parameter value [25]. We then further investigated this behavior by decreasing SIRT1 activity by reducing *VSIRT1c (non-histone deacetylation activity)* and *VSIRT1d (histone deacetylation activity)* to determine if either of these parameters would result an increase of CRY/PER mRNA levels. Similar to Asher et al., a decrease in *VSIRT1c* results in CRY/PER mRNA level decreases, as shown in Fig. 7. Similar to Nakahata et al., a decrease in VSIRT1d results in an increase of CRY/PER mRNA levels, as shown in Fig. 8, due to a smaller repressive effect by SIRT1 on transcription [15,16]. Fig. 9 shows the percentage change in maximal levels of CRY/PER mRNA levels over the parameter values that exhibit stable oscillations for the SIRT1-related parameter values. We find these results to be robust by reducing each of these three parameters to 30% of the original value (this is near the lower limit where parameter decreases for *VSIRT1c*, *VSIRT1d*, and *VNADc* continue to result in oscillations) and conducting a sensitivity analysis as described above. Sensitivity analysis for each of these parameters shows increases in the maximal levels of CRY/PER mRNA consistently for *VSIRT1d* and decreases for both *VSIRT1c* and *VNADc*. While both *VSIRT1c* and *VSIRT1d* parameters contribute to the overall state of the system, the parameters *VSIRT1c* and *VSIRT1d* have opposing effects and parameter *VSIRT1c* has a stronger overall effect within the model.

### Simulating the effect of DNA damage on circadian rhythms

Next, we examined the effect of DNA damage on circadian rhythms, which has been experimentally studied by Oklejewicz, et al. using Rat-1 fibroblasts [11]. In the current model we have examined this effect via the two possible mechanisms. First, the current model allows the examination of DNA damage as simulated by the activation of CHK2 (*kchk2*) to phosphorylate PER monomer and dimer that triggers their degradation, and the second being sharp decreases in NAD levels on the circadian clock using changes in *kPARP* to simulate PARP1 activity. As a major participant in DNA damage response, PARP1 activity becomes greatly increased in response to DNA strand breaks and is recruited to the sites of DNA damage in a matter of minutes [20]. Since ionizing radiation results primarily in phase advancement, we asked whether perturbations in PARP1, singly or in combination with CHK2, could produce similar phase responses, and if so by what mechanism these phase advancements arise.

To compare the phase responses between simulations, we use the ratio of the maximum phase advancement in a PRC to the maximum phase delay in the PRC [28]. The PRC for the Hong 2009 is described in Fig. 2 of Hong et al. [28]. For comparison, Table 4 shows these PRC ratio results for both the Hong 2009 model using the current model and re-parameterized (using the parameters from Table 2) and for the current model under various parameter conditions. Additionally, in Table 4 we provide the fraction of the area under the PRC that is positive; these values are largely consistent with the ratio metric. With the re-parameterized model, we first perturb the model using the same *kchk2* (*kchk2* = 0.2) from Hong et al. There is a discrepancy in values for the ratio (3.54 as originally published versus 3.0193 here), but we believe this may be a by-product of numerical analysis and we use our value as the point of comparison. Perturbing the current model using the same *kchk2* (*kchk2* = 0.2) value results in a larger positive fraction of the area under the phase response curve.

**Fig 10:**
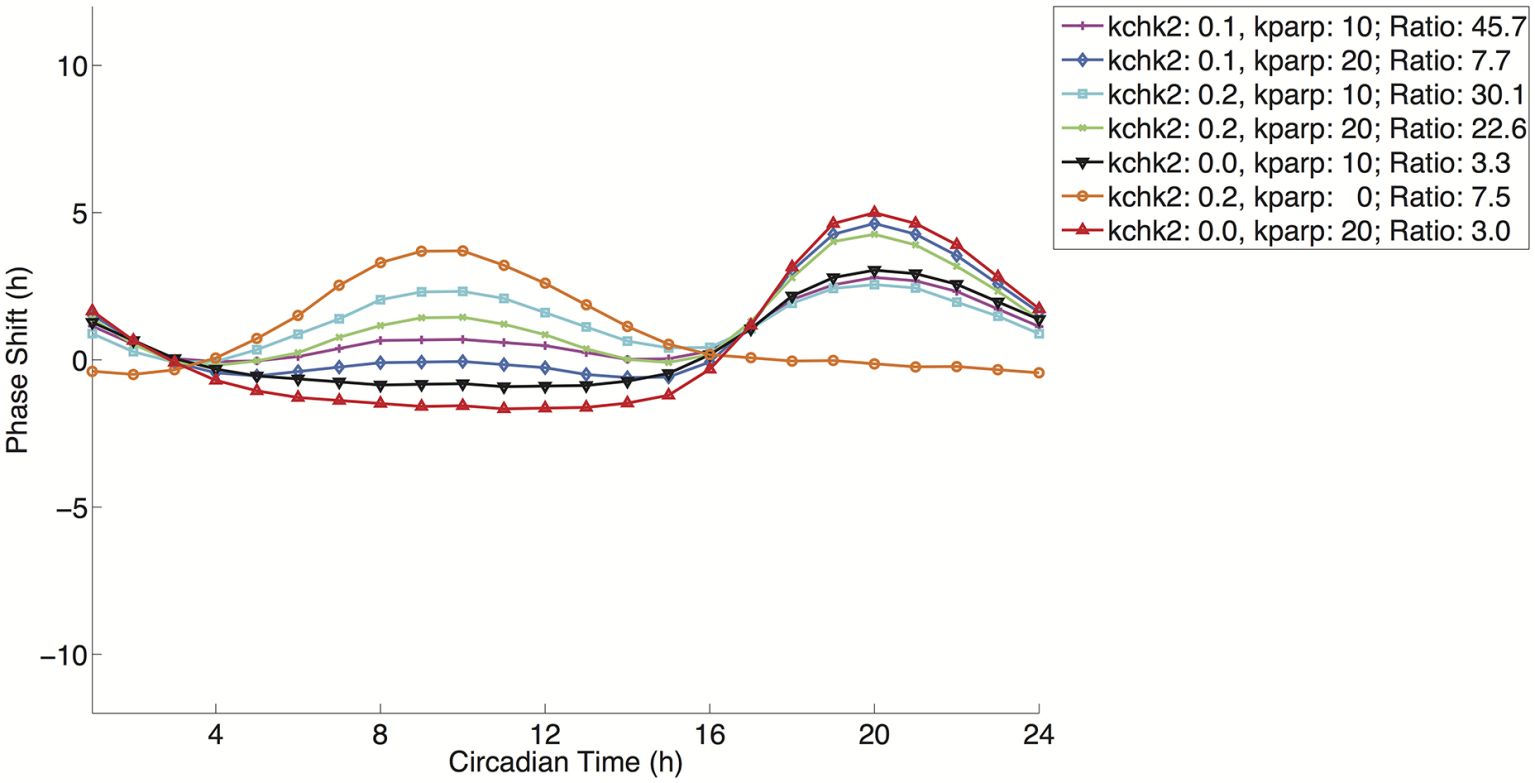
PRCs for various parameter combinations of kchk2 and kparp. Magenta/Plus: Advance to Delay Ratio: 45.7412; Light Blue/Diamond: Ratio: 7.6848; Cyan/Square: Ratio: 30.1066; Green/X-Mark: 22.6498; Black/Down Triangle: Ratio: 3.3353; Orange/Circle: Ratio: 7.5161; Red/Up Triangle: Ratio: 3.007. Details on parameter values used are found in Table 4.

We next calculated the positive area fraction using only *kPARP* (*kPARP* = 20) for a treatment duration of two hours. This yielded a PRC where the majority of the area was positive, similar to the one observed for the re-implemented Hong 2009 model; 0.6235 versus 0.8513, respectively. We next wondered whether a combination of perturbations would yield a larger positive area fraction. Using the values kchk2 = 0.1 and kparp = 10, we calculated a positive area fraction slightly greater than the fraction value for the CHK2 perturbation alone in the current model. This is with a CHK2 value of half the value used for the Hong 2009 re-parameterization. At kchk2 = 0.2 and kparp = 20, we produce a positive area fraction that is almost completely positive. These results are robust when we conduct a sensitivity analysis at these high levels of perturbation with parameter changes of 20%. The model is less robust to changes in parameters that further decrease NAD production; this is expected given the strain on NAD levels due to PARP activity. The resulting PRC has a near bimodal appearance. Within the context of the model this effect has a direct relation on the activities of SIRT1 in the model both as an inhibitor of transcription and as a mechanism for the destabilization of PER protein. This effect of this CHK2 perturbation occurs at a circadian time of 10 hours, shown in Fig. 10, which is during peak of PER dimer levels (the dominant form of the repressor in the system), shown in Fig. 2A. This degradation allows mRNA levels of PER and NAMPT to rise in advance of the unperturbed model thereby resulting in a strong phase advancement. The delays for this CHK2-dependent PRC occur at troughs of PER dimer levels. This degradation of the PER dimer repressor at this point causes a slight increase in the maximum PER mRNA level relative to the unperturbed model in the subsequent circadian cycle resulting in the delay observed in the CHK2-dependent PRC.

The CHK2-dependent PRC is in contrast to the PARP-dependent PRC, shown in Fig. 10, at the highest value tested (*kparp* = 20). At this value, a Type 1 PRC is also produced, but whereas the CHK2 perturbation degrades PER dimer levels, the simulated consumption of NAD by PARP removes an inhibitory effect (the deacetylation of PER leading to its degradation by the activity of SIRT1) on this repressor causing an opposite effect; the peak of the PARP-dependent PRC occurs at roughly circadian time 20 hours and its trough at circadian time 10 hours. This increase in PER dimer levels causes an inactivation of transcription by repressing the activity of BMAL1/CLOCK, which acts as both the transcription factor complex and as the histone acetylatransferase in the model. Therefore, these two perturbations, NAD depletion and PER degradation, may have different effects depending on the circadian time. The disparate effects of these two perturbations are seen in Fig. 10; advance-delay ratio and positive area fraction results are listed in Table 4. In combinations of the two perturbations, a bimodality in the PRC emerges at larger values of the two perturbations, which is not directly seen experimentally in the observations by Oklejewicz et al. suggesting that if this is a mechanism that exists biologically, then the balance between these two forms of perturbation may be under additional regulation [11]. Yet, the phase response curves seen experimentally in response to DNA damage are undoubtedly the products of several forms of perturbation each that may have a dominant effect depending on the phase of the system during perturbation.

## Discussion

Here we have developed a simple model that expands on the work of both Hong et al. and Smolen et al. to produce a mathematical model that connects circadian rhythms to DNA damage response and metabolism via the regulation of chromatin remodeling [28,31].

The current model predicts a molecular mechanism through which multiple forms of perturbation, as a result of DNA damage, and multiple post-translational modifications can reproduce the experimentally observed phase response curve as shown in Oklejewicz et al. in Fig. 1 of that publication [11]. We began with the hypothesis that the activities of SIRT1 and PARP1 in regulating the circadian rhythm could impact on the primarily phase advancement seen in circadian oscillations during the response to genotoxic stress given their known interactions with core circadian clock components. To investigate this question, we expanded a previous model to account for the activity of SIRT1 in the regulation of transcription and circadian clock components and the activity of PARP1 during DNA damage response. The model reveals that the regulation of the circadian clock may be wired in a way that integrates multiple forms of post-translational modifications as a mechanism to respond to environmental stress; in the case of acetylation, this post-transcriptional modification is controlled using a circadian feedback mechanism through regulation of NAMPT. We examined phase response curves resulting from various conditions by using the simulated effects of CHK2 and PARP1 activity. The results of our *in silico* study help to confirm the potential for CHK2 involvement in producing the experimentally observed PRC in the presence of an autocatalytic positive loop regulating PER. Our model suggests that additional regulatory mechanisms may factor into the observed PRC. The expanded model shows that NAD depletion via PARP1 activity may produce a similar PRC result as experimentally observed through the removal of the SIRT1 inhibitory effect. Models with both NAD depletion and CHK2 activity reproduce the observed PRC best. This raises the possibility, that multiple perturbations may work in concert to produce the observed PRC.

The current model also addresses an apparent contradiction in the literature as to the effect of SIRT1 inhibition on the levels of PER2 mRNA [15,16,25]. We showed that differential SIRT1 activity targeting specifically either histone or non-histone component deacetylation may account for this contradiction. Alternatively, this contradiction may suggest an additional mechanism that was not controlled between the two sets of experimental observations and may also be missing from the current model. A recent publication by Xydous et al. has suggested that the byproduct of the SIRT1 activity, nicotinamide, (though not specific to its reaction and not accounted for in the current model) may be able to affect histone methylation levels leading to alterations in gene expression in a manner that is independent of SIRT1 activity [40]. Though the inclusion of histone methylation is outside the scope of the current model, this is a potential avenue for future examination and may further add to our understanding of how multiple post-translational modifications are co-regulated to affect circadian activity.

One part of the SIRT1-PARP1 system that obviously remains to be explored through a more comprehensive model would include a more complete description of the salvaging of NAD, including the activity of NMNAT1 that yields an intermediate step in this process. Although, NAMPT is the rate-limiting step in the salvage process, it catalyzes the first step in the conversion of nicotinamide (the by-product of SIRT1 and PARP1 catalysis) into nicotinamide mononucleotide; a substrate that is subsequently converted into NAD by NMNAT1 [17]. In the current model, only NAMPT has been included, because it is under circadian control, and because it is known to be rate limiting in the production of NAD. Yet several publications have shown that SIRT1 can bind to nicotinamide mononucleotide adenylyltransferase 1 (NMNAT1), and it has been hypothesized that this activity may help to stimulate SIRT1 activity [41]. This would be an interesting next step to pursue, as well as the more detailed PARP1 dynamics that account for the negative feedback cycle in these dynamics due to its auto-modification capability [20].

As the underlying mechanisms regulating the circadian clock become better understood with respect to the effects of post-translational modifications, such as acetylation, methylation, sumolyation, and ubiquitination, the addition of these factors can be used to refine current models of circadian rhythms.

## Supporting Information

S1 File Model in the Systems Biology Markup Language (SBML) format generated using COPASI (http://www.copasi.org). (XML)
